# Retraction: Seed priming with growth regulators modulates production, physiology and antioxidant defense of Indian squash (*Praecitrullus fistulosus*) under semi-arid conditions

**DOI:** 10.1371/journal.pone.0274231

**Published:** 2022-09-14

**Authors:** The PLOS ONE Editors

The *PLOS ONE* Editors retract this article [1] because it was identified as one of a series of submissions for which we have concerns about authorship, competing interests, and peer review. We regret that the issues were not addressed prior to the article’s publication.

RQ, MES, Atique-ur-Rehman, AR, HMRJ, MAN, and RA-Y did not agree with the retraction. SK and JA either did not respond directly or could not be reached.
